# Idle peripheral intravenous cannulation: an observational cohort study of pre-hospital and emergency department practices

**DOI:** 10.1186/s13049-021-00941-y

**Published:** 2021-08-28

**Authors:** Hugo Evison, Amy Sweeny, Jamie Ranse, Mercedes Carrington, Nicole Marsh, Joshua Byrnes, Claire M. Rickard, Peter J. Carr, Gerben Keijzers

**Affiliations:** 1 Queensland Ambulance Service, GPO Box 1425, Brisbane, QLD 4000 Australia; 2 Department of Emergency Medicine, Gold Coast University Hospital, 1 Hospital Boulevard, Southport, QLD 4215 Australia; 3 Department of Emergency Medicine, Robina Hospital, 2 Bayberry Lane, Robina, QLD 4226 Australia; 4 Alliance for Vascular Access Teaching and Research, Menzies Health Institute Queensland, Griffith University, G40 Griffith Health Centre, Level 8.86 Gold Coast Campus Griffith University, Southport, QLD 4222 Australia; 5 School of Nursing and Midwifery, Griffith University, N48 Health Sciences Building, Level 2.06, 170 Kessels Road, Southport, QLD 4111 Australia; 6 School of Medicine, Griffith University, Teaching Griffith Health Centre - G40 Gold Coast Campus Griffith University, Southport, QLD 4222 Australia; 7 Faculty of Health Sciences and Medicine, Bond University, 14 University Dr, Robina, QLD 4226 Australia; 8 Nursing and Midwifery Research Centre, Royal Brisbane and Women’s Hospital, Level 2 Building 34 Royal Brisbane and Women’s Hospital, Herston, QLD 4209 Australia; 9 Centre for Applied Health Economics, School of Medicine, Griffith University, N78 Sir Samuel Griffith Building, Level 2.11, 170 Kessels Road, Southport, QLD 4111 Australia; 10 School of Nursing and Midwifery, National University of Ireland Galway, 26 Upper Newcastle, Galway, H91 E3YV Ireland; 11 Herston Infectious Diseases Institute, Metro North Hospitals and Health Service, Herston, QLD 4006 Australia; 12 School of Nursing Midwifery and Social Work, The University of Queensland Centre for Clinical Research, Herston, QLD 4006 Australia

**Keywords:** Cannula, Emergency department, Idle, Pre-hospital, Vascular access device

## Abstract

**Background:**

Unused *('idle')* peripheral intravenous catheters (PIVC) are those not used within 24 hours of insertion. There is little data on cannulation practices and idle PIVC rates in emergency settings, especially the pre-hospital environment.

**Methods:**

This was an observational cohort study set in south-east Queensland, Australia using data from a large tertiary level emergency department (ED) and the local statutory ambulance service. Demographic, clinical and PIVC data were collected over two periods; 9 February–18 March 2017 and 5 January–4 February 2018. Adult patients were included if they were allocated an Australasian triage scale (ATS) category between 2 and 5, and had a PIVC inserted in the pre-hospital setting or ED. PIVC use was defined as idle if no fluids, medications or contrast were administered intravenously within 24 hours of insertion. Comparisons between pre-hospital and ED practice and idle PIVC status were undertaken using descriptive statistics and logistic regression.

**Results:**

A total of 1249 patients with a PIVC (372 pre-hospital; 877 ED) were included. Overall, 366 PIVCs (29.3%; 95% CI 26.9%–31.9%) remained idle at 24 hours. In the pre-hospital group, 147 (39.5%) PIVCs inserted were not used pre-hospital, and 74 (19.9%) remained idle. In comparison, 292 (33.3%) PIVCs placed in the ED remained idle. ED staff more frequently inserted PIVCs in the antecubital fossa than paramedics (65.5% vs. 49.7%), where forearm PIVC insertion was more common pre-hospital than in ED (13.7% vs. 7.4%). Nursing staff inserted idle PIVCs at a rate of (35.1%) compared to doctors (29.6%) and paramedics (19.9%). Having a PIVC inserted in the ED was the only factor significantly (*p* ≤ .001) predicting an idle outcome (Odds Ratio: 2.4; 95% CI 1.7–3.3).

**Conclusion:**

One-third of PIVCs inserted within the emergency setting remained idle, suggesting unnecessary risk and costs. Pre-hospital and ED PIVC insertion practices differed, with idle PIVCs 2.4 times more prevalent if inserted in the ED than pre-hospital and with greater use of antecubital insertion. Reasons for these differences are not well understood and requires more targeted research.

## Background

Establishing peripheral intravenous catheter (PIVC) access is a fundamental element for facilitating modern day emergency health care [1]. It allows for the administration of a variety of symptom-relieving and/or potentially lifesaving infusates [2]. The ubiquitous use and perceived low risk associated with this device has led to frequent and routine insertion [3]. However, PIVC insertion is described as a traumatic, invasive procedure, with over half of patients finding the procedure at least moderately painful [4]. As 12–25% of first time attempts are unsuccessful [5, 6], the possible distress caused by failure of the first attempt is an important consideration in the clinical decision making whether a PIVC should be inserted.

Inflammatory complications are common, and studies have reported that 18–54% of PIVCs are associated with phlebitis [6–10]. Serious infections can occur as the PIVC acts as a potential conduit for the introduction of infectious pathogens [11]. Previous research examining rates of intravascular related bloodstream infections (BSIs) found a pooled mean of 0.1% PIVC BSIs per 100 devices [12]. *Staphylococcus aureus* bacteraemia carries the highest morbidity and mortality rate of healthcare associated infections and is associated with up to 25% of all PIVC related bloodstream infections [13, 14]. The prolific use of the device implies many patients are suffering serious adverse events [15].

A fundamental concept when performing any procedure, including PIVC insertion, is to ensure potential benefits outweigh the risks. There are a wide range of clinical indications for PIVCs, making appropriate patient selection for insertion challenging. Although the clinical need for a PIVC is sometimes obvious, many are inserted in haemodynamically stable patients without a clear or immediate requirement. It could be hypothesised that junior clinicians may be tempted to insert a PIVC for the purpose of training or skills maintenance. Similar could be said for experienced clinicians who have seen the rapid and unexpected deterioration of patients. Clinicians may insert a PIVC ‘*just in case,*’ with negligible potential benefits, and studies report up to 50% remain idle and are never used [16, 17].

Peripheral intravenous catheter insertion in the ED has been associated with higher rates of idle PIVCs [17–19]. The Cannulation Rates in the Emergency Department Intervention Trial (CREDIT) study conducted in Queensland, Australia highlighted that one-third of PIVCs inserted in the ED were idle [20]. Reducing rates of idle or never used PIVCs should be a goal of all emergency health care systems, including the pre-hospital setting. Paramedics are an important group of providers who are skilled in PIVC insertion and are commonly the first point of healthcare contact for the patient. Sparse knowledge of PIVC insertion practices in the pre-hospital environment exists [21].

The circumstances under which paramedics insert a PIVC and the number of attempts taken to complete the procedure have not been comprehensively explored within existing literature. Extrication difficulties, patient movement, bystanders and weather conditions are factors which may negatively impact successful PIVC insertion. The austere nature of pre-hospital care may lead to different decisions on when, or why, to insert a PIVC compared to clinicians in other settings such as the ED. This may have an effect on the proportion of idle PIVCs compared to ED insertion. The lack of literature regarding rates of idle PIVCs, especially in the pre-hospital setting has been highlighted previously [21].

We aimed to describe contemporaneous PIVC insertion practices in pre-hospital and ED patient cohorts, with a focus on idle PIVC rates. Specifically our objectives were to: (1) describe and compare the rate of idle PIVCs between the pre-hospital and ED setting; (2) compare pre-hospital and ED cannulation practices, and (3) identify patient and clinician factors associated with idle PIVCs.

## Methods

### Design and setting

This was an observational cohort study in south-east Queensland, Australia, studying adult patients from both a large tertiary level emergency department (ED) and the local statutory ambulance service who had a PIVC inserted. Human research ethics committee approval, including waiver of consent, was obtained. The ED is a mixed adult and paediatric Level 1 trauma centre with over 110,000 ED presentations annually.

### Participants

**Fig. 1:**
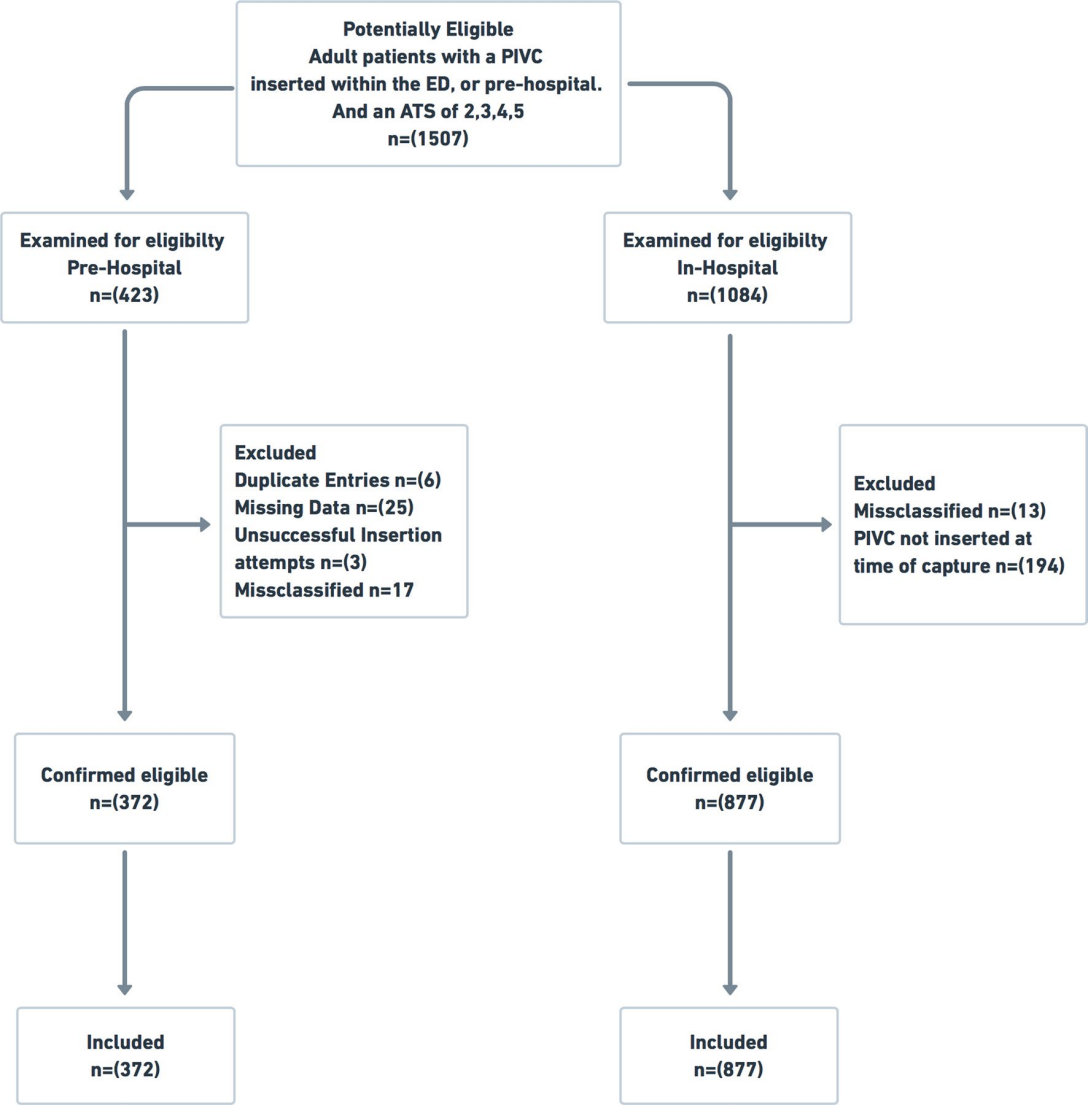
Patient flow. PIVC: Peripheral intravenous catheter, ED: Emergency Department, ATS: Australasian triage scale

During 9 February–18 March 2017 and 5 January–4 February 2018, data on 1507 eligible ED presentations were prospectively collected by a research nurse [22]. Inclusion criteria were patients who were adults and either had a PIVC inserted within the ED, or by a paramedic in the pre-hospital setting. The Australasian triage scale (ATS) ranges from 1 to 5, with 1 representing the most urgent category [23]. Patients who had an ATS category of 1, had a PIVC inserted in another hospital and were transferred between hospitals were excluded (Fig. 1).

### Primary outcome measure

The primary outcome measure was the idle PIVC rate, defined as the proportion of PIVCs inserted but not used within 24 hours of placement. PIVC use was defined as administration of intravenous medication, fluid or contrast. PIVC use for a ‘flush dose’ of normal saline to ensure cannula patency and/or drawing blood from a PIVC for pathology was not defined as PIVC use. Other outcomes of interest included comparison of PIVC insertion practices including PIVC size, anatomical insertion site and inserter staff level, as well as identifying predictors of idle PIVCs.

### Data sources

For participants who were prospectively identified as having a PIVC inserted by the ambulance service, the electronic ambulance report form (eARF), ED and inpatient electronic medical records (EMRs) were retrospectively interrogated for relevant variables. From these sources, data on PIVC use within 24 hours were extracted and included: patient demographics (e.g. gender, age), PIVC characteristics (e.g. gauge, insertion site) and inserting clinician characteristics (e.g. designation, years of service) were collected (Table 1). For participants who had a PIVC inserted within the ED, the same features were extracted from an existing prospectively captured data set [22] (Table 2).

**Table 1:** Data captured from electronic ambulance report forms (eARFs)

Patient features	PIVC insertion features	Clinician features
Demographics, age, sex	Anatomical insertion site	Years of service
Presenting complaint	Cannula size/gauge	Clinicians scope of practice*
Triage category	Number of insertion attempts	Clinician medal number
Distance from scene to hospital (kms)	Insertion success (yes/no)	
Time from scene to hospital (mins)	Complications^#^	
Time on scene (mins)	PIVC use (yes/no and if yes, what was it used for?)	
Time of day		

^*^Clinicians scope of practice refers to the paramedic’s clinical level, paramedics included in the study were student paramedics, advanced care paramedics, critical care paramedics and High Acuity Response (HARU) paramedics

^#^Arterial puncture/fluid extravasation/haematoma or haemorrhage/venous air embolus

**Table 2:** Data captured from ED medical records

PIVC insertion features	Patient features	Clinician features
Anatomical insertion site	Demographics, age, sex	Doctor
Cannula size/gauge	Presenting complaint	Nurse
Number of insertion attempts	Triage category	
Insertion success (yes/no),	Time of day	
Complications		
PIVC use (yes/no and if yes, what used for)		

### Sample size and data analysis

We calculated the sample size necessary to identify a clinically significant difference in idle PIVC rates between pre-hospital and ED settings. Based on existing literature showing the idle PIVC rate to be around 30%, we considered a difference of ± 10% to be clinically significant [20]. In order to be powered at 80% with an alpha of 0.05, we required data on 390 patients with PIVC insertions in both pre-hospital and ED groups.

Data analysis involved both univariate and multivariate methods, using SPSS v26.0. Simple descriptive statistics (proportions, frequencies, and means of central tendency) were calculated. A 95% confidence interval around the proportion of "idle" PIVCs was calculated using statistical software (Open Epi), and the Wilson score method. Comparisons of proportion of idle PIVCs, and insertion practices between pre-hospital and ED practice employed chi-square tests. Univariate associations of potential predictors of idle PIVCs were examined in the pre-hospital setting, and across the emergency setting. Crude odds ratios (OR) and 95% confidence intervals (CI) were calculated.

Two logistic regression models using a backwards conditional approach were developed with the dependent variable of idle PIVCs. The first assessed predictors of idle PIVCs for pre-hospital PIVCs only, with independent variables of age, sex, gauge, insertion site, cannulator characteristics, time (minutes) at site, time of day, distance from site to hospital, and time from scene to hospital (Table 1). The second assessed predictors of idle PIVCs for all PIVCs placed in the emergency setting (pre-hospital and ED combined), with a more limited set of potential predictors that were applicable to both settings (age, sex, cannulator experience, presentation time of day, gauge, anatomical insertion site and insertion setting). The pre-hospital specific variables, such as time on scene were not included. The ATS for the pre-hospital group was determined from the ED ATS. The number of records with missing data was presented for each variable. In all inferential univariate comparisons, the missing data category was excluded from the chi-square tests. In the multivariable modelling, records missing potential predictors were automatically excluded case-wise from the initial backwards conditional analysis. Variables that remained significant in the backwards model were then forced into a new model with only those variables. Adjusted odds ratio (OR) and 95% confidence interval (CI) were calculated for this final model.

## Results

The eligible patients and relevant exclusions are outlined in Fig. 1. Of the 1249 patients with PIVC insertions included (372 pre-hospital and 877 in ED), nearly half (45.2%) were over 60 years of age and half (50.1%) were female. Table 3 describes the characteristics of PIVCs placed in the ED, pre-hospital setting and combined, where Table 4 describes the pre-hospital setting only.

**Table 3:** Characteristics of PIVCs placed in the ED, pre-hospital setting and combined

Characteristic of cannulation	All emergency PIVCs (n = 1249)		ED (n = 877)		Pre-hospital (n = 372)		*p *value
	n	valid %	n	%	n	%	
**Use within 24 hours**							
Used within 24 hours	883	70.7	585	66.7	298	80.1	< 0.001
Idle	366	29.3	292	33.3	74	19.9	
**Arrival mode of patient**							
Ambulance	674	54.0	302	34.4	372	100.0	N/A
Self-presented	575	46.0	575	65.6	0	0.0	
**Clinician inserting**							
Doctor	277	22.2	277	31.6	NA		
Nurse	587	47.0	587	66.9	NA		N/A
Paramedic	372	29.8	NA		372	100.0	
Unknown	13	1.0	13	1.5	0	0.0	
**Seniority of first clinician cannulating**							
Student	8	0.6	5	0.6	3	0.8	Not assessed
Level 1	68	5.4	20	2.3	48	12.9	
Level 2	997	79.8	741	84.5	256	68.8	
Level 3	113	9.0	98	11.2	15	4.0	
Unknown	63	5.0	13	3.5	50	13.4	
**Patient factors**							
**Age**							
18–29	184	14.7	141	16.1	43	11.6	0.117
30–59	500	40.0	344	39.2	156	41.9	
60 +	565	45.2	392	44.7	173	46.5	
Sex							
F	626	50.1	462	52.7	164	44.1	0.005
M	623	49.9	415	47.3	208	55.9	
**Triage category**‡							
1	0	0.0	0	0.0	0	0.0	< 0.001
2	503	40.3	316	36.0	187	50.3	
3	661	52.9	492	56.1	169	45.4	
4	74	5.9	67	7.6	7	1.9	
5	3	0.2	2	0.2	1	0.3	
Missing	8	0.6	0	0.0	8	2.2	
**Time category**							
Night (18:00–05:59)	308	24.7	201	22.9	107	28.8	0.012
Day (06:00–17:59)	941	75.1	676	77.1	265	70.4	
**Number of attempts at cannulation**‡							
1	988	79.1	684	78.0	304	81.7	< 0.001
2	140	11.2	118	13.5	22	5.9	
3 +	83	6.6	75	8.6	8	2.2	
Missing	38	3.0	0	0.0	38	10.2	
**Cannula insertion site (first)**‡							
Antecubital fossa	759	60.8	574	65.5	185	49.7	< 0.001
Dorsum	270	21.6	175	20.0	95	25.5	
Forearm	116	9.3	65	7.4	51	13.7	
Unknown	104	8.3	63	7.2	41	11.0	
**Cannula gauge (first) (n = 328**)‡							
Other (14 or 16 g)	4	0.3	1	0.1	3	0.8	< 0.001
18 g	286	22.9	139	15.8	147	39.5	
20 g	842	67.4	675	77.0	167	44.9	
22 g	51	4.1	40	4.6	11	3.0	
Unknown	66	5.3	22	2.5	44	11.8	
**Purpose of PIVCs use in first 24 hours (one only)**							
Multiple non-pathology purposes	426	34.1	320	36.5	106	28.5	
Other single purpose only	139	11.1	137	15.6	2	0.5	
Fluids only	130	10.4	105	12.0	25	6.7	
Pain only	83	6.6	23	2.6	60	16.1	
Nausea only	32	2.6	Not captured				
					32	8.6	
Used, but specific use not collected^	73	5.8	NA	0.0	73	19.6	
Pathology only or no use identified	366	29.3	292	33.3	74	19.9	
**Purpose of PIVC use includes (multiple options available)**							
Pain	280	22.4	129	14.7	151	40.6	< .001^
Fluids	413	33.1	365	41.6	48	12.9	< .001^
Other IV	550	44.0	437	49.8	113	30.4	< .001^
Pathology	843	67.5	843	96.1	0	0.0	
Unsure	73	5.8	0	0.0	73	19.6	

Five attempted PIVCS not included

^‡^ Unknown/missing data excluded from inferential statistics

^Compares ED vs pre-hospital used for this purpose vs without this purpose. Student (either setting), Level 1 (graduate paramedic, intern), Level 2: ACP II, RN, resident, Level 4: CCP, HARU, CN, CNC, consultant or registrar

**Table 4:** Characteristics of PIVCs placed pre-hospital and factors associated with pre-hospital idle PIVCs

	Total (n = 375)		PIVC used (n = 298)		Idle PIVC (n = 74)		p-value	Crude OR (95% CI)
	n	%	n	%	n	%		
**Age**								
18–29	43	11.5	34	79.1	9	20.9	0.867	1.2 (0.7–2.0)
30–59	158	42.1	127	81.4	29	18.6		1.0 (0.4–2.3)
60 +	174	46.4	137	79.2	36	20.8		1.0 (reference)
**Sex**								
F	165	44.0	131	79.9	33	20.1	0.922	1.0 (0.6–1.7)
M	210	56.0	167	80.3	41	19.7		1.0 (reference)
**Triage category‡**								
1	0	0.0	0	NA	0	NA	0.122	Not calculated
2	188	50.1	150	79.8	37	19.8		
3	171	45.6	134	45.0	35	20.7		
4	7	1.9	7	100.0	0	0.0		
5	1	0.3	0	0.0	1	100.0		
Missing	8	2.1	7	87.5	1	12.5		
**Presenting complaint**								
Chest Pain	109	29.1	82	76.6	25	23.4	Not calculated	Not calculated
Pain	75	20.0	69	93.2	5	6.8		
Neurological	54	14.4	41	75.9	13	24.1		
Trauma	40	10.7	31	77.5	9	22.5		
Other	34	9.1	21	61.8	13	38.2		
Dyspnoea	27	7.2	25	92.6	2	7.4		
Gastro-intestinal	13	3.5	13	100.0	0	0.0		
Seizure	13	3.5	9	69.2	4	30.8		
Diabetic Related	5	1.3	4	80.0	1	20.0		
Sepsis	5	1.3	3	60.0	2	40.0		
**Complaint category**								
Pain or chest pain	184	49.1	151	83.4	30	16.6	0.119	1.0 (reference)
Any other	191	50.9	147	77.0	44	23.0		1.5 (0.9–2.5)
**Time category‡**								
Night (18:00–05:59)	108	28.8	89	83.2	18	16.8	0.346	1.0 (reference)
Day (06:00–17:59)	264	70.4	206	78.6	56	21.4		1.3 (0.7–2.4)
**Time on scene ‡ (minutes)**								
< 15	35	9.3	27	79.4	7	20.6	0.663	1.2 (0.5–3.2)
15–29	214	57.1	167	78.0	47	22.0		1.3 (0.7–2.3)
30 +	115	30.7	93	82.3	20	17.7		1.0 (reference)
Missing	11	3.2	11	91.7	0	0.0		
**Time in transit ‡ (depart site to ED triage in minutes)**								
< 15	116	30.9	95	81.9	21	18.1	0.257	1.0 (reference)
15–29	197	52.5	157	80.5	38	19.5		1.1 (0.6–2.0)
30 +	53	14.1	37	71.2	15	28.8		1.8 (0.9–3.9)
Missing	9	2.4	9	100.0	0	0.0		
**Total time pre-hospital (minutes) ‡** **(n = 366)**								
< 30	31	8.3	24	77.4	7	22.6	0.152	0.9 (0.3–2.7)
30–44	154	41.1	117	76.0	37	24.0		1.0 (0.5–2.1)
45–59	129	34.4	109	86.5	17	13.5		0.5 (0.2–1.1)
60 +	55	14.7	42	76.4	13	23.6		1.0 (reference)
Missing	6	1.6	6	100.0	0	0.0		
**Distance to hospital (kms) ‡**								
< 10	184	49.1	146	80.2	36	19.8	0.978	1.0 (reference)
10–19	124	33.1	98	79.7	25	20.3		1.0 (0.6–1.8)
20 +	63	16.8	51	82.3	12	19.0		1.0 (0.5–2.0)
Missing	4	1.1	3	75.0	1	25.0		NA
**Number of attempts ‡ (n = 334)**								
1	307	81.9	249	81.9	55	18.1	0.806	Not calculated
2	22	5.9	19	86.4	3	13.6		
3 +	8	2.1	7	87.5	1	12.5		
Missing	38	10.1	23	60.5	15	39.5		NA
**Insertion site (first) ‡**								
Antecubital fossa	185	49.3	155	83.8	30	16.2	0.689	1.0 (reference)
Dorsum	95	25.3	76	80.0	19	20.0		1.3 (0.7–2.4)
Forearm	51	13.6	41	80.4	10	19.6		1.6 (0.6–2.8)
Missing	44	11.7	26	59.1	15	34.1		
**Gauge (first) ‡**^								
16 g	3	0.8	1	33.3	2	66.7	0.141	1.0 (reference)
18 g	147	39.2	124	84.4	23	15.6		1.1 (0.6–2.0)
20 g	167	44.5	136	81.4	31	18.6		
22 g	11	2.9	9	81.8	2	18.2		
Missing	47	12.5	28	59.6	19	40.4		
**Skillset ‡**^**#**^ **(first cannulator)**								
ACP II	256	68.3	214	83.6	42	16.4	0.551	Not calculated
CCP	14	3.7	11	78.6	3	21.4		
Graduate Paramedic	48	12.8	36	75.0	12	25.0		
HARU	1	0.3	1	100.0	0	0.0		
Student	3	0.8	3	100.0	0	0.0		
Missing	53	14.1	33	62.3	17	32.1		
**Years of service ‡** **(first cannulator)**								
< 2 years	67	17.9	53	79.1	14	20.9	0.15	0.9 (0.4–1.9)
2–4 years	57	15.2	52	91.2	5	8.8		0.3 (0.1–0.9)
5–9 years	94	25.1	79	84.0	15	16.0		0.6 (0.3–1.3)
10 + years	100	26.7	77	77.0	23	23.0		1.0 (reference)
Missing	57	15.2	37	64.9	17	29.8		

Three unsuccessful cannulations are included in the totals column only

^‡^unknown/missing category not included in inferential statistics. ^comparing 20–22 vs ≤ 18 g

^#^ACP II = Advanced Care Paramedic level 2, CCP = Critical Care Paramedic, Graduate Paramedic = paramedic during their internship, HARU = High Acuity Response Paramedic, Student = student paramedic on university placement, ED = Emergency Department

### Idle PIVC rates

The overall idle PIVC rate in the emergency setting (pre-hospital and ED combined) was 29.3% (n = 366), 95% CI: 26.9%–31.9% (Table 3). Of the 372 patients who had their PIVC inserted prehospitally, 1 in 5 (19.9%, n = 74) had an idle PIVC, whereas one-third (33.3%, n = 292) of the patients who had a PIVC in ED had an idle PIVC. Nearly 40% (39.5%, n = 147) of PIVCs inserted in the pre-hospital setting were not used by paramedics whilst in the ambulance. Nursing staff were most likely to insert a PIVC that remained idle (35.1%, n = 206) compared to doctors (29.6%, n = 82) and paramedics (19.9%, n = 74) (Table 5)*.*

**Table 5:** Characteristics of PIVCs inserted (ED and pre-hospital combined) and factors associated with idle PIVCs

Characteristics of PIVC	All emergency PIVCs (n = 1249)		Used within 24 h (n = 883)		Idle (n = 366)		p-value	Crude OR (95% CI)	Adjusted OR (95% CI)
	n	valid %	n	%	n	%			
**Geographical location of insertion**									
Pre-hospital	372	29.8	298	80.1	74	19.9	< 0.001	1.0 (reference)	1.0 (reference)
ED	877	70.2	585	66.7	292	33.3		2.0 (1.5–2.7)	2.4 (1.7–3.3)
**Staff type**									
Nurse	587	47.0	381	64.9	206	35.1	< 0.001	2.5 (1.8–3.5)	Not tested in model
Doctor	277	22.2	195	70.4	82	29.6		2.0 (1.3–2.9)	
Paramedic	372	29.8	298	80.1	74	19.9		1.0 (reference)	
Other ED staff	13	1.0	9	69.2	4	30.8			
**Arrival mode of patient**									
Pre-hospital	674	54.0	504	74.8	170	25.2	0.001	1.0 (reference)	Not tested in model
Self-presented	575	46.0	379	65.9	196	34.1		1.5 (1.2–2.0)	
**Seniority (first clinician)**									
Student	8	0.6	7	87.5	1	12.5		Not calculated	Not tested in model
Level 1	68	5.4	50	73.5	18	26.5			
Level 2	997	79.8	702	70.4	295	29.6			
Level 3	113	9.0	82	72.6	31	27.4			
Unknown	63	5.0	42	66.7	21	33.3			
**Age**									
18–29	184	14.7	120	65.2	64	34.8	0.05	1.4 (1.0–2.1)	Not retained in model
30–59	500	40.0	351	70.2	149	29.8	0.33	1.1 (0.9–1.5)	
60 +	565	45.2	412	72.9	153	27.1	0.13	1.0 (reference)	
**Sex**									
F	626	50.1	438	70.0	188	30.0	0.571	1.1 (0.8–1.4)	Not retained in model
M	623	49.9	445	71.4	178	28.6		1.0 (reference)	
**Triage category ‡€**							0.04	0.8 (0.7–1.0)	0.7 (0.5–0.8)
1	0	0.0	0	NA	0	0.0			
2	503	40.3	340	67.6	163	32.4			
3	661	52.9	477	72.2	184	27.8			
4	74	5.9	57	77.0	17	23.0			
5	3	0.2	2	66.7	1	33.3			
Missing	8	0.6	7	87.5	1	12.5			
**Time category**									
Night (18:00–05:59)	308	24.7	232	75.3	76	24.7	0.04	1.0 (reference)	Not retained in model
Day (06:00–17:59)	941	75.3	651	69.2	290	30.9		1.4 (1.0–1.8)	
**Number of attempts ‡**									
1	988	79.1	690	69.8	298	30.2	0.163	Not calculated	Not tested in model
2	140	11.2	107	76.4	33	23.6			
3 +	83	6.6	63	75.9	20	24.1			
Missing	38	3.0	23	60.5	15	39.5			
**Cannula insertion site ‡ (first)**									
Antecubital fossa	758	60.8	525	69.3	233	30.7	0.049	1.5 (0.9–2.6)	1.6 (1.0–2.6)
Dorsum	269	21.6	195	72.5	74	27.5		1.8 (1.1–2.9)	1.1 (0.8–1.5)
Forearm	116	9.3	93	80.2	23	19.8		1.0 (reference)	1.0 (reference)
Unknown/other	106	8.3	70	66.0	36	34.0			
**Cannula gauge (first) ‡^ (n = 328)**							0.039		
Other (14 or 16 g)	4	0.3	2	50.0	2	50.0		1.0 (reference)	Not retained in model
18 g	286	22.9	218	76.2	68	23.8			
20 g	842	67.4	584	69.4	258	30.6		1.4 (1.0–1.9)	
22 g	51	4.1	37	72.5	14	27.5			
Unknown	66	5.3	42	63.6	24	36.4			

Five attempted PIVCS not included

^‡^Unknown/missing category not included in inferential statistics

€Triage category treated as an ordinal variable, classified as 2, 3 and 4 or 5

^comparing 20–22 vs ≤ 18 g.Variables marked as not retained in model dropped out of the multivariable model after adjustment for predictors with greater estimates of effect. Variables marked as not tested were not tested in the model as they were not comparable across the ED and pre-hospital setting, did not make clinical sense as a predictor, or were highly correlated with variables already in the model. Student (either setting), Level 1 (graduate paramedic, intern), Level 2: ACP II, RN, resident, Level 4: CCP, HARU, CN, CNC, consultant or registrar

### Comparison between pre-hospital and ED

Table 3 outlines the comparison between pre-hospital and ED for PIVC insertion. Women represented a smaller proportion of PIVCs placed by pre-hospital clinicians (44.1% vs 55.9%). In addition, PIVCs placed pre-hospital were more likely to be in more urgent ATS categories, with 50.3% from ATS 2. While PIVCs placed within the ED included fewer patients with ATS 2 (36.0%) and more ATS 3, 4 and 5 compared to pre-hospital PIVCs. For PIVCs inserted in the ED 96.1% were used for pathology, with 33.3% used only for pathology (or no use identified). Although both ED and pre-hospital clinicians inserted most PIVCs in the antecubital fossa, pre-hospital staff were more likely to use the forearm (13.7% vs 7.4%, (*p* < 0.001). Pre-hospital staff documented fewer attempts at cannulation (range 1–4, 9.0% with two or more attempts), compared to ED (range 1–10, 22% with two or more attempts), (*p* < 0.001). No PIVC complications were recorded on ambulance eARFs.

### Pre-hospital PIVC

The most common reasons PIVCs were placed pre-hospital were chest pain and other types of pain (Table 4). Of those 372 PIVCs successfully placed pre-hospital, 225 (60.5%) were used by the ambulance service, mostly for administration of pain relief (n = 151). Approximately half (n = 112, 49.8%) of all patients who had their PIVCs used pre-hospital had intravenous anti-emetics administered through them.

### Predictors of idle PIVC

Within the pre-hospital cohort a logistic regression multivariable model did not find any significant predictors of idle PIVCs amongst the characteristics studied, including distance or time to hospital (Table 4). Factors univariately associated with increased idle PIVC rate for the emergency setting (pre-hospital and ED combined) are outlined in Table 5 and included; PIVCs insertion in ED, by doctors or nurses, patients who self-presented, insertion during the day (0600–1759 h) and insertion in places other than the forearm*.* In the multivariable logistic regression analysis, ED PIVC insertion (OR 2.4, 95% CI 1.7–3.3) and triage category (ATS 2 and 3 vs 4 and 5) (OR 0.7, 95% CI 0.5–0.8), were the only factors significantly predicting idle PIVCs (Table 5).

## Discussion

Across the emergency setting, including pre-hospital and ED, nearly 3 in 10 (29.3%) PIVCs placed remained idle. ED clinicians were 2.4 times more likely than paramedics to insert a PIVC that was not used within 24 hours of insertion. These findings suggest that paramedics in our study made sound decisions on when to insert a PIVC compared to hospital staff. Although this left 19.9% of pre-hospital PIVCs that were not used within the first 24 hours, our results for pre-hospital PIVC insertions align with recent literature that advocates for clinicians not to insert a PIVC unless they are over 80% certain it will be used [20]_._

We also demonstrated that a substantial proportion (39.5%) of PIVCs inserted by paramedics were unused while in the pre-hospital setting. Comparatively, we report a lower percentage of unused pre-hospital PIVCs than previous studies which report rates as high as 72–83% [24, 25]. The rate of unused pre-hospital PIVCs in this study is of importance and prompts questions surrounding paramedics’ motives for inserting pre-hospital PIVCs. Further investigation is warranted to achieve a comprehensive understanding of pre-hospital decision making regarding PIVC insertion.

The provision of symptomatic treatment in the pre-hospital environment is an essential aspect of patient care. Although analgesia provision is common and evidence-based [26], the role of antiemetics for treating nausea via the intravenous route is not as well defined [27]. We found that pre-hospital PIVCs were most frequently used for analgesia and treating nausea. Approximately half (49.8%) of all PIVCs inserted and used pre-hospital had intravenous anti-emetics (mainly ondansetron) administered through them; we therefore hypothesise that a number of these PIVCs were inserted solely for the purpose of administering anti-emetics. Since October 2017, paramedics in our setting have obtained access to ondansetron oral disintegrating tablets in addition to the long-standing availability of intramuscular anti-emetics. It is possible that a subset of PIVC insertions for administering anti-emetics were clinically not indicated, and even unnecessary.

For paramedics and ED staff in our study, the clinical guidelines for PIVC insertion are broad, and rely largely on clinician discretion. The number of PIVC insertion attempts differed between the pre-hospital and ED healthcare providers. Emergency department practitioners recorded more and a wider range of attempts than paramedics with ranges of 1–10 and 1–4 attempts respectively. Clinical guidelines for paramedics in our study recommend that a maximum of three PIVC insertion attempts are permitted per officer. It is likely that these guidelines, transport factors and time constraints consistent with the pre-hospital environment contributed to lower numbers of insertion attempts by paramedics. Conversely, many more attempts were taken for patients within the ED requiring intravenous access; this may be due to an emergent requirement for further diagnostic imaging or pharmacology.

Peripheral intravenous catheters inserted within the ED were frequently used for obtaining blood samples (96.1%) and in one-third of the patients, the PIVC was used for blood sampling only. This study excluded pathology collection as an appropriate PIVC use due to the procedure carrying increased risks to the patient and a higher rate of complications when compared with venepuncture [28]. It is likely that patients, especially with less urgent ATS categories within our sample had a PIVC inserted in the ED solely for the purpose of pathology collection. The large proportion of PIVCs being used for pathology collection in our study is congruent with other studies [16, 29]. Egerton-Warburton et al. reported a multimodal approach aimed at reducing inappropriate PIVC insertions [29]. This approach was successful in reducing unnecessary PIVCs, but not in lowering rates of PIVCs inserted for pathology collection only [29]. The financial burden and increased patient risks of PIVC placement as opposed to venepuncture must be carefully considered prior to deciding which procedure is to be performed. Previous research estimates that costs associated with PIVC insertion and removal range between A$13-$22; this does not include the costs related with the ongoing maintenance of the device [20, 30]. More importantly, patients with a PIVC are at continued risk of developing complications. These patients are also more likely to receive intravenous therapy that may not be appropriate [31]. It is predictable that these patients will have higher financial costs compared to those who receive venepuncture. Although pathology collection using a PIVC may be reasonable if intravenous medication, fluids or contrast is required, a PIVC for the sole use of specimen collection can lead to downstream adverse events [31]. Our study defined an idle PIVC as unused within 24 hours of insertion, excluding a normal saline flush or the collection of pathology via the PIVC. Previous studies have adopted various definitions of an idle PIVC; however, the most frequently used definition is consistent with ours, namely a PIVC that was inserted and remained unused [32]. It can be argued that clinically indicated PIVCs may become idle due to rapid changes in the patient’s clinical condition or haemodynamic status. The complexities of patient physiology may create uncertainty around the definition of an appropriate PIVC and a zero incidence of idleness is unrealistic. This imprecision has resulted in the absence of an accepted definition of a clinically indicated, yet unused PIVC. As such, we deemed any idle PIVC in our study as an inappropriate insertion. A recent systematic review highlighted the lack of literature surrounding the rate of idle PIVCs in the pre-hospital setting [21]. Our study is the first study to directly compare idle rates of PIVCs between pre-hospital and ED settings. This work demonstrates current practices of PIVC insertion across the broader emergency setting and lays foundations for future work about the clinical decision making of clinicians around PIVC insertion.

### Limitations

This was a single setting observational study and as a result, data may not be generalisable with other populations. While the ED PIVC data was prospectively collected, pre-hospital predictor variables were retrospectively collected. Documentation of PIVC attempts is mandatory in both the pre-hospital and ED environments; however, inaccuracy in these records may be present, such as the number of recorded attempts before successful insertion. The lack of complications recorded from pre-hospital PIVCs may also reflect this. Some unmeasured variables may have had an effect on the difference in idle PIVC rates. We did not collect any data on the reasoning of the PIVC inserting clinician, limiting our ability to comment on the proportion of idle PIVCs which may have been clinically indicated and appropriately inserted.

## Conclusion

The rate of idle PIVCs within the broader emergency health care setting in this study was 29%. This study provides insight into pre-hospital and ED PIVC insertion practices and highlights the differences in rates of idle PIVCs between both cohorts, with PIVCs inserted in the ED 2.4 times more likely to remain idle compared to those inserted pre-hospital. Reasons for the differences in these practices are not well understood and requires more targeted research. We recommend a qualitative approach with clinicians of different backgrounds and experience to capture a more in-depth insight into clinician perceptions regarding PIVC insertion and use, guided by the findings of this study. Reducing the rate of idle PIVC insertion will lead to less risk and discomfort for patients and cost savings for healthcare systems; as such this should be the goal of all healthcare providers.

## Data Availability

The datasets generated and/or analysed during the current study are not publicly available due to local ethics and governance regulations but are available from the corresponding author on reasonable request.

## Abbreviations

PIVC: Peripheral Intravenous Cannula
ED: Emergency Department
ATS: Australasian Triage Scale
BSI: Blood Stream Infections
CREDIT: Canulation Rates in the Emergency Department Intervention Trial
eARF: Electronic Ambulance Report Form
EMR: Electronic Medical Records
